# Exploring housing policies in five Swedish municipalities: alternatives and priorities

**DOI:** 10.1186/s12889-022-12672-5

**Published:** 2022-02-08

**Authors:** Christina Heller, Lisa Ekstam, Maria Haak, Steven M. Schmidt, Björn Slaug

**Affiliations:** 1 Department of Health Sciences, Faculty of Medicine, Lund University, Lund, Sweden; 2 Department of Nursing Education and Integrated Health Sciences, Faculty of Health Sciences, Kristianstad University, Kristianstad, Sweden

## Abstract

**Introduction:**

Housing shortage due to population growth within metropolitan areas, combined with an ageing population, has put pressure on current housing provision in Sweden. Thus, there is an urgent need to develop sustainable housing policies to accommodate the growing number of seniors in accessible home environments. This study aimed to gain an in-depth understanding of how municipalities currently address housing accessibility issues and to explore what types of policy solutions they consider for the future.

**Material and methods:**

Five Swedish municipalities were selected to represent a diversity of the population, housing provision approaches, and geographical areas. To understand current housing policies, two key actors (e.g. public officials, housing adaptation grant managers, city architects, etc.) from each municipality participated in semi-structured interviews (*N* = 10). Subsequently, those key actors, two senior citizens, and three researchers participated in a research circle to explore future policy solutions. Data were analyzed using content analysis.

**Results:**

The interviews revealed common approaches to deal with housing accessibility issues such as regular renovations and maintenance, individual adaptations based on specific needs, and seeking collaboration with private housing actors on housing provision matters. Possible measures suggested for the future included increasing the national coordination of housing accessibility policies, amending legislation to only allow the construction of housing according to strengthened accessibility standards, and introducing economic incentives for seniors to move from housing with poor accessibility to more accessible accommodations.

**Conclusions:**

Municipalities struggle with the lack of accessible and affordable housing for their ageing population, despite a large variety of policies from economic incentives to research and development policies. The results suggest that collaboration needs to be improved between all actors involved in housing policies. Preventive measures within the current laws may be needed to strengthen the construction of more accessible and affordable housing for populations ageing in place.

## Background

The European countries are all facing a structural housing shortage due to strong population growth within metropolitan areas and large cities [1, 2]. The biggest share of the European population resides in cities (40.4%) as well as towns and suburbs (31.6%) [3]. Low property values in areas outside of growth regions have created a high-risk situation for developers to produce new housing in these areas, which has been reported particularly in Luxemburg, Germany, Ireland, the Netherlands, and Sweden [1, 4].

Furthermore, the current demographic change is a challenging phenomenon as low birth rates and a higher life expectancy transit the population structure towards a much older population, and will therefore sooner or later affect all European countries. The proportion of individuals aged 80 years or older is expected to increase from 5.9% in 2020 to approximately 9.1% in 2040, and the expected increase is 11.1% in 2050 [5]. Hence, the ageing population, as well as the accompanying demographic change, will put financial pressure on old-age support systems as well as on housing provision policies when it comes to accessible housing development [6].

Moreover, the ageing of the population in many European countries has resulted in policymakers giving more attention to how to improve and develop sustainable approaches to meet the needs of senior citizens. For instance, to accommodate the desire of an ageing generation that wants to live in their homes as long as possible, “ageing in place” has become a major policy strategy in many countries and is defined as “remaining living in the community, with some level of independence, rather than in residential care” [7]. From a societal perspective, “ageing in place” can be supported by measures taken to design or adapt the physical home environment in a way that enables older people to maintain independence in activities of daily living and thereby potentially decrease public expenditures through less need for institutional care and home services [8]. The physical environment is a central health determinant and previous as well as recent research shows that the housing environment may impact older people’s autonomy [9], independence [10], and participation [11]. Moreover, there is evidence that living in an inappropriately designed housing environment may increase risks of fall accidents [12], poor mental health [13], and mortality [14]. Housing policies can therefore be instrumental in addressing several major public health issues.

Although all European countries have similar difficulties to meet the needs of senior citizens, housing policies differ widely both with regard to how they are realized and the culture in which they emerge. The Swedish public housing system stands out among the countries within the European Union, even if it is sometimes considered as an example of “social housing” [2, 15]. There is no generally agreed-upon definition of social housing, but a recent literature review suggested that social housing is a system that provides long-term housing to a group with limited financial resources, utilizing a distribution system and subsidies [16]. The housing systems in many European countries include policies that meet this definition of social housing. For instance, in Germany “publicly subsidized housing” for vulnerable household groups such as single parents, people with lower incomes, or migrants is common [15]. In Denmark, to take another example, social housing is provided by non-profit housing associations owned and organized by its members but regulated by the state. Although the Swedish system includes subsidies to low-income and other vulnerable groups, in general, a queue system based on time and housing availability is applied, and there is no special housing market for low-income or socially disadvantaged households. While not social housing according to the definition suggested by Granath Hansson and Lundgren [16], the Swedish public housing market is characterized by the societal responsibility of the municipalities [17] to plan and provide adequate and affordable housing for all citizens [18, 19]. Unlike the other European countries, Sweden also has an association of public housing companies, Public Housing Sweden; the Swedish name is “Allmännyttan”, which literally means “public utility” or “for the benefit of everybody” [15, 20]. However, at the same time as these public housing companies have a societal responsibility, they are required to be business-oriented and make profit, which in reality may be difficult to combine [21].

The policy choices of the different countries may also reflect different value systems and ideologies, as well as the supply and demand of public and social housing. As an example, Germany is characterized by the principle of subsidiarity and relies in the first instance on the individual as well as on family responsibility. Only in the second instance on solidarity in society as a whole, that is, on state support [22]. Concerning housing policies, an important turning point in Germany in the 1980s was the abolition and marketization of nonprofit housing companies which resulted in a general decrease in social housing. The amount of social housing is only about 4% of the total housing stock today and should mainly support individuals with a lower income [23]. Denmark, in comparison, is a country with a long tradition of social renting that has not registered a decrease in the share of social housing out of the total housing stock since the early 2000s and represents a generally high share of social accommodation with 21% of the total housing stock [1, 2]. In Sweden, the association of public housing companies has been instrumental in the implementation of housing policies. At present, approximately 50% of all rental apartments are owned by public housing companies. Particularly in the 1940s and 1950s, there was overcrowding and a big shortage of accommodations in Sweden, which gave rise to the “Million Homes Program” that was introduced in the early 1960s. The objective was to build multi-family dwellings in a high quantity and speed [24] nationwide. The Swedish societal planning of the housing market is organized on three different levels. The national building targets are set out in the budget that the government submits to the Parliament while the County Councils (21 in total) are responsible for the regional spatial planning and the Municipalities (290 in total) are working with the physical execution which includes location and physical appearance of buildings [25].

To accommodate the growing number of senior citizens, housing needs to be accessible, since multimorbidity, functional decline, and dependence on mobility devices [26] tend to increase in older age and make it more onerous to reach objects, move around in the environment and manage everyday life [27]. According to the current Swedish Planning and Building Act (PBL) new housing must be accessible in its design and technical characteristics for individuals with functional limitations [28]. However, environmental barriers (e.g. wall-mounted cupboards and shelves placed extremely high or high thresholds and/or too high steps at the entrances) in or outside the homes are common in the existing Swedish housing stock and hinder older people and people with functional limitations from using their homes adequately [29]. Furthermore, recent research expounded that a lack of stair handrails at entrances was associated with increased mortality risk [14]. Additionally, a previous Swedish governmental report identified three major issues in Sweden. First, approximately 50% of inhabitants aged 65 years or older live in single-family houses where the bathroom is installed on the second floor, which leads to difficulties for individuals with functional limitations to use their sanitation facilities. Second, 50% of all accommodations built before the 1970s are still in use, but before the late 1970s, no law considered lifts for buildings with more than one floor. Third, an absence of maneuvering space for a rollator or wheelchair within ordinary housing is a major challenge for people with functional limitations and disabilities, since they have difficulties moving within or entering/exiting their homes [4].

Addressing the challenges created by the housing shortage in metropolitan areas and rapidly ageing populations within the ordinary housing stock are also topics of intense interest in worldwide politics and Public Health research. An important aspect of appropriate housing for older people is the extent to which the physical housing environment facilitates or impedes activities of daily living when people age and their functional capacity is declining, that is, how accessible the housing environment is [30]. For instance, there is sufficient maneuvering space while using a walking device, kitchen cupboards are placed at a height easy to reach, or entrance doors stay in the open position. While some studies focused on housing accessibility in this sense [29, 31] as well as on housing adaptations [11, 32–34], other research has concentrated on older people’s preferences and experiences [35]. Also, the economic perspective [36] and outcomes such as fear of falls [37] or quality of life [32] are topics that get particular attention in current studies. Although the research on housing accessibility has been recognized as important, there is a paucity of studies that focus on examining the actual policies of municipalities addressing current and future housing needs. As policies at the municipal level play an important role in achieving overarching political goals, it is important to understand the processes and reasoning behind them to serve policymakers to make the most informed decisions and thereby improve the housing situation and health of the population. Thus, this study aimed to gain an in-depth understanding of how municipalities currently address housing accessibility issues and to explore what types of policy solutions they consider in the future to better support housing accessibility for an ageing population.

## Methods

### Design and setting

This study has a qualitative and participatory approach. As a first step, to gain a deeper understanding of current housing policies we conducted individual interviews with key actors such as development strategists, housing adaptation grant managers, etc., involved in shaping or executing housing policies at the municipal level. Considering such aspects as previous collaborations and commitment to engage in research, five Swedish municipalities (from a total of 290) were included in the study: Eslöv, Perstorp, Vänersborg, Örebro and Östersund [38] (Table 1). These municipalities were purposefully selected to reflect diversity in size, geographic location, socio-economic conditions, and ethnicities. By doing so, we intended to capture a variety of perspectives, experiences, and visions for the future, rather than to compare the policies of the municipalities. Additionally, we conducted a document analysis of the municipalities’ housing supply plans, to increase our knowledge on future housing policies targeting the older population. In a second step, we actively engaged the same key actors in a research circle with two separate online meetings, where they were instructed to suggest and prioritize between alternative policy options for the future.

**Table 1:** Municipality characteristics^a^

Municipality	County	Population size	Density (inhabitants/km^**2)**^	Annual median income in Euro (2019)^b^	Proportion of foreign-born inhabitants (%)
Eslöv	Skåne	34,321	81.4	26,783	19.7
Perstorp	Skåne	7465	47.1	22,255	26.4
Vänersborg	Västra Götaland	39,671	61.5	26,764	15.3
Örebro	Örebro	156,170	113.9	27,089	18.3
Östersund	Jämtland	64,194	29.0	27,365	9.4

^a^Statistics Sweden [38]

^b^SEK was converted to EURO using the exchange rate on 13 September 2021 (1 SEK = 0,099 Euro. The annual median income in 2019 in Sweden was 28,215 Euro)

### Participant characteristics

To cover different areas of responsibility, we interviewed key actors with a variety of professional and administrative positions. Ten key actors (two from each municipality) participated in the individual interviews and ten in the research circle. One participant (P 01) dropped out after the interview due to a job switch and was replaced by another key actor (P 11) from the same municipality and with a similar position when the research circle was conducted (see Table 2). The key actors (with the replacement, six men and five women) were 28-63 years of age (median = 43 years), Swedish speaking, and had on average 8 years of working experience within the municipality. Diversity in terms of sex, age, and professional positions was strived for. Two senior citizens (one man, one woman, aged 74 and 86) from a national pensioner organization were invited to the research circle to add sentiments and perspectives of older people on future housing accessibility policies*.*

**Table 2:** Participant characteristics

Participant no.	Sex	Professional position	Participation in:	
			Individual interview	Research circle
P 01	Man	Development Strategist	x	
P 02	Woman	Housing Adaptation Grant Manager	x	x
P 03	Woman	Chief of Social Services Department	x	x
P 04	Man	City Architect, Planning, and Construction Manager	x	x
P 05	Man	Program Director Social Welfare	x	x
P 06	Man	Group Leader, Housing Adaptation Grants	x	x
P 07	Woman	Mission Strategist in the Health and Care Administration	x	x
P 08	Woman	Land and Development Administrator	x	x
P 09	Man	Development Leader Community Planning	x	x
P 10	Man	Housing Adaptation Grant Manager	x	x
P 11	Woman	Development Strategist		x
P 12	Woman	Senior Citizen		x
P 13	Man	Senior Citizen		x

We followed the principles of the Helsinki Declaration, and each participant provided written informed consent. The study was approved by the Swedish Ethical Review Authority (No. 2020-01643).

### Data collection

#### Interviews

Nine interviews were conducted online via a video conferencing tool between June 2020 and January 2021 and one interview was conducted in person as physical distancing could be guaranteed. All interviews were conducted by L.E. and C.H. together, and took approximately 40-60 min each. In preparation for the individual interviews, a compilation of the demographic, political, and organizational structure of each municipality was gathered. The municipalities’ housing supply plans were also read before each interview, to get a broad overview of current strategies for housing accessibility. Before the interviews, an information letter and a letter of consent were sent to the participants. We followed an interview guide but sometimes modified the questions during the interview to be compliant with the specifics of the interviews. All interviews were audio-recorded and transcribed verbatim by C.H. and a professional transcribing company. C.H. conducted a quality check on the transcripts to ensure consistency between audio recordings and transcripts and the removal of all personal identifiers. Three co-authors have Swedish as their native language while two are bilingual in Swedish with English and German as their native languages.

#### Policy documents: housing supply plans

To complement the data on current and future housing policies, we utilized the publicly and freely available housing supply plans from each of the five municipalities, which were recommended by the key actors and found on their websites.

#### Research circle

In spring 2021 M.H., B.S., and C.H. conducted an online research circle with two meetings 14 days apart, and with an average length of 3 h. A research circle is a collaborative method that is developed as a Participatory Action Research approach and has its origin in the Swedish study circle tradition [39]. The purpose of a research circle is to elicit new ideas to influence or change a specific situation together with researchers and participants from different backgrounds [39, 40]. Together with researchers, the key actors from the five municipalities and two senior citizens were engaged in a joint effort to develop new knowledge and ideas for alternative policy solutions.

As a starting point in the first meeting, we presented and discussed the preliminary results of the analysis from the individual interviews, focusing on current interventions and policy solutions to address housing accessibility issues for an ageing population. Hence, we instructed the participants to discuss the following question: “If you could change anything concerning housing accessibility for the aging population, what would you change or improve?”. The proposals were collected and summarized by the researchers and sent out to the participants prior to the second meeting. As a take-home task, the participants were asked to process and discuss the list of proposals with colleagues or peers and thus bring back additional information to discuss. At the second meeting, the participants first shared their reflections after discussing the list with colleagues or peers. Then there was an open discussion engaging all participants about how to prioritize between the different proposals, and sometimes also specifying, broadening, or merging the original proposals. At the end of the second meeting, all the participants agreed on a prioritized list of policy suggestions for the future. The meetings were documented by audio recordings and memos were taken by the researchers during each meeting. It is primarily the list of prioritized policy suggestions that the participants agreed upon that is used for the analysis in this study.

### Data analysis

We used content analysis according to a meticulous procedure described by Mayring [41, 42] to analyze the data from the interviews, the policy documents, and the research circle. To process all the data and facilitate the analytic procedure, the NVivo software (version 12) [43] was used.

#### Interviews

The analysis started with the interviews, and because of the lack of previous qualitative research focusing on the specific aim of this study, an inductive category formation [41, 42] was deemed appropriate. That is, the first and the last author (C.H. and B.S.) read independently through interview transcripts and labeled relevant words, sentences and sections to identify key findings related to the aim. Subsequently, they coded the material individually, line by line, and defined categories from the resulting codes. After reviewing about 10% of the material, the inductively developed categories were subject to a strong inter-coder agreement check. We calculated a percentage agreement rate for the individual codings, by dividing the number of equivalent codes by the total number of codes, which showed an agreement rate of 59%. C.H. and B.S. thereafter discussed the codings and the categories with L.E. and M.H. until disagreements were resolved and consensus reached. C.H. then proceeded with the categories the co-authors had agreed on until 50% of the interviews were coded. Thereafter, a first light inter-coder agreement check was carried out. In this step, C.H., B.S., L.E., and M.H. reviewed the codings and categories and together re-tested if they were still describing the main information of the material. The last 50% of the transcripts were then coded by C.H. and a second light inter-coder-agreement check was conducted involving all five co-authors. Finally, by identifying common patterns of the categories, over-arching themes were formed. To achieve inter-coder reliability and communicative validation, C.H. analyzed the material as a whole, whilst B.S. coded partially. In an iterative process, all the co-authors jointly discussed the categories and themes until consensus was achieved.

#### Policy documents and research circle

The policy documents and the list of prioritized policy suggestions were analyzed subsequently to the interviews, applying the same analytic methodology. The documents and the list of prioritized policy suggestions were separately coded and categorized by C.H. and light inter-coder checks were conducted where all the co-authors reviewed and validated the categories. The documents mainly confirmed the data gained from the previous interviews, but a few codes were added within the analytical process.

## Results

In the interviews, we found that discussions of current and future housing accessibility policies often were intermingled with thoughts and reflections of different factors that influenced, guided, or restrained the municipalities’ choice of action and their room to maneuver when addressing housing accessibility issues. We identified four such main themes concerning factors that influenced current and future policies, i.e., legal, organizational, socio-demographic, and political factors. We present these factors first and then proceed with the findings of actual measures or policies currently carried out or considered for the future.

### Factors influencing housing accessibility policies

#### Legal factors

Policies addressing housing accessibility has to conform to a legal framework and several of the participants emphasized that the Swedish Planning and Building Act [44] and the National Board of Housing, Building, and Planning (BBR) [45] guide their policies regarding housing accessibility on the municipal level. Nonetheless, it was also noted that sometimes the current laws are vague and leave room for interpretation. On the other hand, they recognized there is limited leeway towards private housing companies to demand more than what is regulated in the law, for instance regarding renovation and housing adaptations. An officeholder at a municipal management office formulated it like this in the interview:

*"Yes, and we can not make those demands [to private housing companies]. That is the crux, that we can only make demands that are supported by PBL and BBR, otherwise, there will be special technical requirements. So we can give inspiration to [private housing companies] …, and then we can give tougher directives to the municipal housing company.”*

#### Organizational factors

The influence of different decision levels and the organizational structure within the municipality was mentioned by many participants. For example, issues, where a holistic approach seems appropriate, may instead be handled by committees with more narrow expertise. It was also described how the decision level occasionally leads to conflicts of aims when striving to address housing accessibility issues properly, such as considering individual circumstances that are addressed differently by general policies at the municipal level.

*“So again, they [the Environmental- and Community Building Administration] are at the micro perspective in this particular residential building and in fact have the right competence and better skills, but we [the municipal management office] must always see the bigger picture, the bigger picture, the bigger picture. We can not say "Yes, absolutely on this [one]", because then we lose everything else. It is to both balance, politics, administration, and then also the municipality's offices and administrations. And the same with Health and Social Care where you can think*―*I do not have a great specific example, but they focus more on the micro perspective or on individual matters*―*for example "this person must have a solution in this way". So we may somehow get a policy mix that goes against it [the individual solution], we are not allowed to do it.”*

#### Socio-demographic factors

The growing number of seniors was described as a major factor influencing housing policies both currently and when planning for the future. It was acknowledged that the growing number of seniors should significantly affect the development of new housing as well as the adaptation of the current housing stock. It was also considered a major challenge for the municipalities to predict the needs of the seniors since they are a heterogeneous group with different needs and various levels of functional limitations.

*"It's very difficult to predict, because it could be that all those who turned 80 that we see in the statistics, "oh now there will be 30 new 80-year-olds compared to last year", perhaps they already live in senior housing and then there is no housing issue that needs to be resolved in that way. Or they are super healthy, live alone in a single-family house. In that case there are actually 30 single-family houses where healthy 80-year-olds live, who may actually be able to keep their house with the help of friends and acquaintances. Or they can not do it… So that is how it is, again macro-micro, we see a number. And I think above all it will only increase, the number of older people living in single-family houses will increase more and more. And there is also a bigger discussion here about how to get them to move. That challenge will only get bigger."*

In the interviews, there was also the reasoning of how supply and demand for new housing and high market prices affect the construction of newly built accessible housing. In sparsely populated areas, it was recognized a challenge that there are no market players who want to invest and build. Moreover, the possibilities for private individuals to receive bank credits for new expensive construction is limited, despite the fact that there exists a need for new, more accessible housing.

#### Political factors

Political governance and different political views and values were frequently discussed as important factors influencing how housing accessibility issues are addressed. Many participants expressed that the political governance concerning how housing issues are addressed may not always take fact-based information from the public officials sufficiently into account. Instead, policies and courses of action are decided more from a political and ideological perspective. It was also emphasized that the political color of the governing party or coalition, and their awareness of housing accessibility issues, is crucial for how future policies concerning these matters will evolve.

*“But there is always a big political discussion about what should be built. Should we build single-family houses, should we build rental apartments? If we build rental apartments, what kind of people will move in? If we build single-family houses, who do we get then? So, that can be quite… a discussion that I think can be quite clearly politically based. You have your own political views, you go hard on them, but rarely back them up with arguments that are actually based on facts, or another theory."*

### Current and future housing accessibility policies

**Fig. 1:**
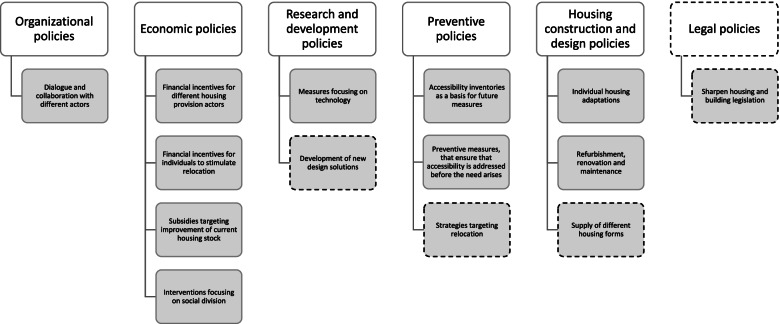
Six main themes with 14 subsumed categories of how municipalities address housing accessibility issues. Themes and categories with dashed frames were mostly considered in terms of future housing accessibility policies

We identified six main themes of housing accessibility policies from the content of the interviews and the policy documents: Organizational policies, Economic policies, Research and development policies, Preventive policies, Housing construction and design policies, and Legal policies. For each theme, we identified one or several categories, see Fig. 1. The results here mainly derive from the interviews. The housing supply plans added some complementary information and mostly from the bigger municipalities, with more elaborated plans.

#### Organizational policies

The theme of Organizational policies has one category focusing on *Dialogue and collaboration with different actors*. The participants described that the municipalities strive to be in dialogue with various actors such as pensioners’ associations, construction companies, carpenters, as well as private and municipal housing companies when they develop their policies.

*"But we try to have a good dialogue [when planning new housing developments]. Now I know that we are only talking about settlements in a development district, but it is still pretty connected anyway, how the accessibility looks on the surrounding streets and other things as well. So in these matters we also have a good dialogue, I think, with our accessibility council which is a municipal council, or an interest organization, or whatever you want to call it. ”*

Some of the participants emphasized that the municipality even collaborates with different actors to develop new ideas and solutions on how housing accessibility can be improved. Dialogue lays the groundwork for collaboration and goes one step further since it involves cooperative projects and a joint effort together with various actors.

#### Economic policies

Economic policies had a rich content and we identified four categories within this theme. *Financial incentives for different housing provision actors,* such as incentives to property owners and housing companies to implement housing adaptations, were considered an important tool to improve housing accessibility. Moreover, it was recognized that public expenditures through home services, for instance, can be decreased if seniors with reduced functional capacity are able to remain living in well-adapted homes that fit their needs, instead of moving to special housing. Furthermore, the participants described *Financial incentives for individuals to stimulate relocation* as something that will grow in importance in the future. The participants also expressed concern, that seniors with functional limitations and lower incomes are more likely to stay in their often inaccessible homes. Financial incentives for those individuals could decrease those “lock-in effects”.

*“And then there are people, I mean older people, who perhaps could actually move to an apartment and leave their house - which is built in the 50s, 60s - but the economic conditions are generally not good for a person living in [this municipality]. So they live [economically] better in their old house rather than moving to something that is perhaps a little more accessible and actually better. And that means that we get very poor mobility in the housing market.”*

The category *Subsidies targeting the current housing stock* captures discussions of the dilemma that although renovations and adaptations of the housing stock would improve housing accessibility, it would also result in higher rents. Especially, it was argued, seniors with lower incomes would have difficulties paying such rents, and property owners, as well as housing companies are therefore hesitant to renovate and improve their accommodations. Currently, there is no financial incentive in the form of subsidies to stimulate the renovation and adaptation of the existing housing stock, which in general is less accessible compared to newly built housing and thus more in need of such measures.

The participants often returned to the topic that housing accessibility issues are very complex and cannot be considered in isolation. In relation to this, *Interventions focusing on social division* were frequently mentioned*.* For instance, measures taking loneliness among seniors into account while building new accessible homes, or social measures such as dealing with homelessness among seniors with substance abuse or poor mental health, by providing them paid accommodation where accessibility issues deserve more consideration than is the case today.

#### Research and development policies

This theme mainly covers discussions related to *Measures focusing on technology*. The participants told about more general ideas of welfare technology and smart homes, but also gave concrete examples. For instance, one participant described the current usage of special assistive technology devices for home assistance personnel, which may compensate for some problems in the physical environment.

Additionally, one participant brought up the *Development of new design solutions* as a suggestion for future policies.

*“Yes, it seems if there could come some new types of lifts that one may not know of or so, that there would be some smart solution to it. //……….//. But there is a company that has a product that is like a staircase, you could say. It looks like a regular staircase. And then you can adapt it so that the stairs, yes, become like a lift, that kind of platform. So it is aesthetically pleasing, but I think it is more perhaps intended for public spaces. I think it's pretty expensive too. But it is possible that there may be some new ideas like that. ”*

#### Preventive policies

*Accessibility inventories as a basis for future measures* were identified by many participants as an essential policy to address housing accessibility issues. Some municipalities had already initialized such inventories while others were involved in discussions on how to go about them in methodological and practical terms. Accessibility inventories were acknowledged as important information to support future measures and initiatives to improve housing accessibility. Most of the actual inventories brought into the discussion, however, were conducted through private or municipal housing companies. Some participants said that even if they saw the benefits of such inventories, they had economic challenges to implement them.

*"In that case, we should… if we were planning to make such an accessibility inventory, then it will still be something where we have to go beyond the regular budget ourselves."*

There were also discussions concerning the benefits of *Preventive measures that ensure accessibility is addressed before the need arises.* One participant specifically mentioned current policies to remove barriers in the homes and make them more accessible in order to prevent falls and injuries in the home environment. To promote good health and sustainable use of economic resources, one municipality’s housing supply plan specifically focused on preventive policies such as social activities, housing counseling, and workshops targeting housing for senior citizens.

Mostly in terms of policies for the future, we identified the additional category *Strategies targeting relocation*. Within the individual interviews as well as in the housing supply plans, there were several examples of incentives of preventive character to stimulate relocation, such as relocation chains, relocation assistance, or information about relocation.

#### Housing construction and design policies

This theme captures one of the principal current policies in all municipalities to address housing accessibility issues, that is through needs-based, publicly funded *Individual housing adaptations.* The most common housing adaptations mentioned in the discussions were removing thresholds, installing and adaptation of showers instead of bathtubs, stair lifts, and stove guards. In general, this policy was positively described. However, the division of responsibility between the municipalities and the regional health care for special housing adaptations was occasionally found to be challenging.

*"I can say, what has happened most about the housing adaptation issue, is that there has been a long dispute between [the municipality] and [the region] about who is responsible for wheelchair garages. Is it a housing adaptation issue or is it the region that is responsible for it, based on the fact that they prescribe the aid? There have been many years of trouble in between."*

In both housing supply plans and interviews *Refurbishment, renovations and maintenance* were described as significant tools to improve housing accessibility as well as social sustainability and equitable living environments*.* The housing supply plans also showed the ambition to meet the needs and wishes of seniors through the *Supply of different housing forms* in the future, including a diversity of tenures such as rental apartments, co-operative apartments, and self-owned single-family houses. Furthermore, in response to increasing social segregation and the complexity of the housing market, we found socially mixed housing with innovative or different housing forms to be promoted. It was also acknowledged that not all older adults can age in place. Some need assistance as soon as functional limitations arise. The municipalities were therefore focusing on building additional intermediate forms of housing such as senior housing and sheltered housing.

#### Legal policies

This theme was mostly discussed in terms of future policies and concerned to *Sharpen housing and building legislation*. There were for instance suggestions by participants to address the challenges created by the vague formulation of the current law, particularly by the mix of accessibility and usability concepts.

*“Even if it [the law] is clear, it becomes unclear precisely in the concept of “accessible and usable”, and it is really like a fluffy cloud to be “accessible and usable”. And the accessibility is easier for them [the building permit officers] to see. Just like yes, there are open areas and there is enough space, there are surfaces to move on, and so on. But the usability is a bit on another level, or how to say. That it is not so easy to get a grip on, I think.”*

### Policy suggestions considered to better support housing accessibility in the future

**Fig. 2:**
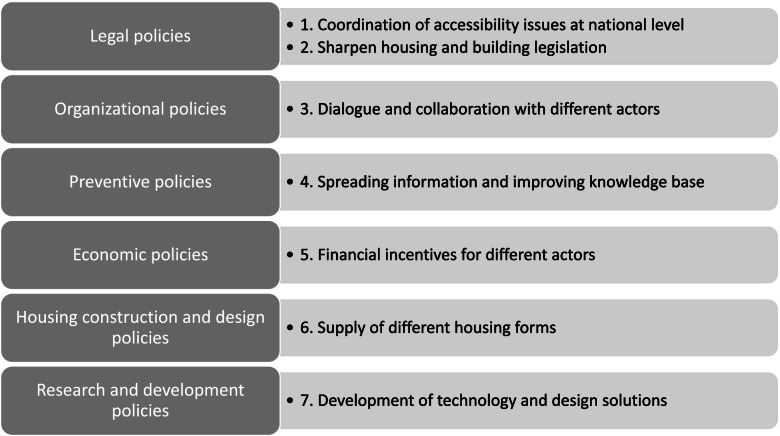
Prioritization of policies considered to better support housing accessibility in the future

Starting from current and future housing accessibility policies and through the joint effort in a research circle format participants from the municipalities, senior citizens, and researchers developed and agreed upon a prioritized list of suggested housing accessibility policies for the future, see Fig. 2.

#### Priority 1

*Coordination of accessibility issues at the national level* was considered to be of the highest priority. This was not mentioned in the interviews or policy documents but was added through the joint discussions of the research circle. The consensus view was that this would create a more comprehensive approach to housing policies, planning, and implementation and strengthen the cross-sectorial collaboration.

#### Priority 2

*Sharpen housing and building legislation* was discussed in the interviews, but the research circle further developed this policy suggestion. It was argued that the current legislation is insufficient regarding universal design and housing accessible for all. Furthermore, it was considered that the current legislation does not sufficiently cover barriers for individuals with cognitive functional limitations, and needs to be amended in this regard. To support the overarching perspective on housing accessibility on a national level, the development of policies that encompass physical safety, fair socio-economic standards, proximity to service, transportation, and culture was embraced.

#### Priority 3

*Dialogue and collaboration with different actors* was prominent already in the interviews as well as the housing supply plans, and the research circle confirmed the importance of such policies. Collaboration and dialogue should include municipal and private housing companies, pensioners, and other non-profit organizations to take different expertise into account and benefit from a diversity of perspectives.

#### Priority 4

*Information and improving knowledge base* among public officials, politicians, and citizens was considered crucial in order to promote improved housing accessibility, and the research circle added more detail compared to what was discussed in the interviews. On an individual level, it was suggested it could be accomplished through for example relocation counselling or apps with support to choose a dwelling that matches individual accessibility needs. Furthermore, the spreading of information and knowledge among politicians to support informed decisions regarding housing accessibility was mentioned. An additional suggestion was to conduct a national information campaign to increase the knowledge and awareness among citizens about the importance to plan for future housing.

#### Priority 5

*Financial incentives* for different actors such as citizens, property owners, housing and construction companies, and municipalities were considered to be of high importance. In addition to incentives mentioned earlier from the interviews, it was argued that municipalities with extra needs due to special socio-demographic circumstances could require long-term national aid packages. Furthermore, national subsidies for targeted measures such as after-installation of lifts were mentioned.

#### Priority 6

*Supply of different housing forms* both regarding building and tenure type was deemed important. In addition to what was brought up in the interviews and housing supply plans, the research circle emphasized that proximity to transportation, service, and culture is of significance. It was therefore argued that when building new housing, areas close to public transport routes should be favoured.

#### Priority 7

*Development of technology and design solutions* to support ageing in place was considered to be highly needed. Such policies were touched upon in the interviews but were further underlined by the research circle. In particular, it was mentioned to be important to develop and implement technical solutions that improve accessibility for individuals with cognitive limitations. The development of innovative technical solutions, such as technical guides in the home environment was also emphasized in order to assure technical accessibility for all.

## Discussion

The study revealed four main factors that were perceived to influence current and future housing policies, described as legal, organizational, socio-demographic, and political factors. Concerning how municipalities currently and in the future deal with housing accessibility issues, six themes were identified. That is, organizational, economic, research and development, preventive, housing construction and design, as well as legal policies. The theme of economic policies appeared to play a central role in how the municipalities handled housing accessibility issues. This theme included, among others, economic incentives to stimulate relocation and subsidies for the renovation of the existing housing stock. To better support housing accessibility for the future, a list of prioritized policies was formulated, where the highest priority was given to legal policies and in particular sharpening building legislation in a manner that puts more specific requirements on building and construction companies to design and build housing that is accessible for all. Coordination of housing accessibility issues at a national level was also considered to be of the highest priority. The participatory approach, where key actors and senior citizens together prepared this priority list of suggested housing accessibility policies for the future, was a novel aspect of the study.

The interviews revealed a common perception that the municipalities are caught in a dilemma between their strategies and goals regarding housing accessibility and the current law as their operational framework. Several participants expressed frustration that they could not require actions by the housing companies that would improve the physical accessibility unless it was clearly supported by the current building legislation. For example, they mentioned how they often struggled with the limited leeway they have towards private housing companies in order to renovate and adjust existing buildings to improve housing accessibility. This reflects the variety and complexity of societal planning problems that municipalities are facing through the involvement of various actors. The complexity of this issue was examined in a recent study, suggesting that the provision of accessible housing for all is a societal issue which by its nature cannot be solved in a way that satisfies all involved stakeholders and actors [46]. Additionally, conflicting interests such as keeping costs down and still providing accessible housing are difficult to balance [47] for the municipalities. In several interviews there was reasoning around the necessity to provide housing of diverse tenure, such as rental apartments, co-operative apartments, and self-owned single-family houses that can attract people with different needs and financial resources. Moreover, the suggestions for interventions focusing on social division and socially mixed housing shows there is pressure on the municipalities and a need to meet the challenges without creating a social housing market for low-income or socially disadvantaged households. The demand for municipal housing companies to adapt to a competitive market and strive for profit makes it difficult to uphold the societal responsibility to vulnerable groups and to realize innovative ideas that may involve economic risks [21].

The interviews showed that legal factors such as the Swedish Planning and Building Act [44] were at the forefront of factors that influence how housing accessibility issues are addressed today. For instance, they argued that barriers that complicate access for persons with cognitive limitations are often overlooked by current legislation and therefore even in newly built dwellings, barriers related to cognitive limitations still occur. However, it was also emphasized in the interviews, that the current building legislation sometimes appears vague and leaves room for interpretations of how housing accessibility requirements should be practically implemented. These results are in line with previous literature that provides examples of how legislation can affect housing accessibility both positively and negatively [47]. Legislation supporting measures for people with functional limitations such as the Swedish Planning and Building Act were generally considered positive by the participants, while negative impacts included the limited leeway towards private housing companies regarding essential renovations and adaptations as well as the vague formulation of the current law in terms of accessibility and usability. These results suggest that political decision-makers at the national level in collaboration with municipalities and other housing provision actors, continuously need to revisit how legislation can be strengthened to support municipalities struggling to enforce housing accessibility policies.

The expressed need to elevate housing accessibility issues to a higher political level than the municipality was a prominent feature of the results, emphasized in the discussions of the research circle. Both the key actors and the senior citizens saw the coordination of accessibility issues at a national level and the sharpening of the housing and building legislation as top priorities, overarching other policies. Especially participants from smaller municipalities expressed, that they needed support and backing from higher national authorities. Earlier research points to the lack of EU regulations on housing accessibility, and this issue remains to be a matter for the national legislation of the EU member states [48]. Initiatives on the EU level could serve to give policies addressing housing accessibility issues further impetus in the member states [49].

Furthermore, how the municipalities addressed housing accessibility in their official policy documents for the ageing population differed. In one of the smaller municipalities, the housing accessibility issues were not evident in the housing supply plans, while the content was richer and more obvious in the plans of the bigger municipalities. However, the participants recognized both in the interviews and the research circle the importance of having policies that are intended to improve housing accessibility. Yet, the participants found the goals set in the policy documents challenging to achieve due to insufficient financial resources or to limited collaboration between municipal committees and other involved actors, e.g. private housing companies. This could indicate a need for policy documents to more clearly outline the resources and conditions needed to fulfill ambitious goals.

The results showed that the level of policy was crucial for implementing adequate measures on a municipal level. Some policies such as renovations of existing buildings and demanding more from private housing companies than what is regulated in the law [4] were constrained by factors on a national level, for instance, the national legislation or economic resources, and were therefore hampering the implementation of measures on the municipal level. As perhaps could be expected organizational factors were considered to influence how housing accessibility issues are addressed. For instance, it was mentioned that issues managed by the social welfare committee tended to apply more of an individual perspective, while issues managed by the building committee tended to apply more of a municipal perspective. As indicated by a previous study, the formal hierarchical structure of bureaucracy can create challenges that decrease information-sharing activities within the organization [50]. Acknowledging the presence of such conflicting perspectives should stimulate key actors to strengthen dialogue and collaboration with each other.

### Strengths and limitations of the study

Although this study did not aim to make any comparisons between municipalities, it should be noted that the challenges within the municipalities e.g. the increasing amount of senior citizens, as well as the current and future policies appeared to be similar across the municipalities. The findings may not reflect a national view on housing policies of the 290 Swedish municipalities. However, they might be an indicator for current and future housing accessibility policies and priorities for the future on a national level, since we purposefully selected the municipalities to reflect diversity in size, geographic location, socio-economic condition as well as ethnicities. Moreover, we strived to cover different areas of responsibilities while including key actors with a variety of different professional and administrative positions, to increase generalizability.

As a consequence of the Covid-19 restrictions, the interviews and the research circle were mainly conducted online. Not meeting in person and using digital communication might on the one hand have impacted the interview situation and conversation to be more efficient and to the point, but on the other hand, may have kept some participants from speaking more freely. However, we were able to gain rich content through the online meetings. To strengthen the study, we included participants that represented a gender-balanced sample with a variation of professional position, and we applied a participatory approach by the inclusion of senior citizens and the use of the research circle methodology. Furthermore, due to Covid-19 restrictions, we could not visit the municipalities to gather information about their physical appearance, governance, and administration, which otherwise would have enriched our results with observations.

Several measures were taken to address the trustworthiness of this study. To ensure credibility, investigator and method triangulation was used. Investigator triangulation was met through the independent coding by two researchers focusing on the same material, as well as the inter-coder-agreement checks within the analysis [42]. By gathering data from different data collection methods such as individual interviews, document analysis as well as research circle, method triangulation was addressed. To apply a member check, the priority list was sent out to the participants of the research circle after the first meeting for feedback and adjusted after discussions. By providing information about the context and research process of this study, we aimed to increase transferability [51].

### Implications

Overall, similar topics were raised in the interviews, policy documents, and the research circle. Our results suggest that collaboration and sharing knowledge between different policy levels within a municipality but also on a national level from one municipality to another would be beneficial to target housing accessibility issues in a joint effort and make informed decisions. Extensive measures such as after-installation of lifts can be economically challenging. However, it might benefit many, such as older people and families with children, and therefore lead to long-term societal gains in terms of increased participation, autonomy, and quality of life in these groups. Moreover, effective communication of the results of existing research on housing accessibility may help key actors in the municipalities to understand areas of concern, such as barriers in the housing environment that increase the risk for falls, and how these can be addressed. Whilst housing accessibility is a complex matter, future studies should focus on the impact of both neighborhood and housing environment in creating supportive environments for the ageing population, in order to improve the health of the population.

## Conclusions

Municipalities struggle with the lack of accessible and affordable housing. Despite a large variety of policies from economic incentives to research and development policies, it seems insufficient to meet the needs of the ageing population. This study contributes to the scholarly literature with insights into the challenges municipalities are faced with and highlights the complexity of the field of housing accessibility, as experienced and formulated by municipal key actors themselves. Even though this study is conducted in a Swedish context, the results have implications for international housing accessibility issues, for instance, preventive measures such as strengthening the individual knowledge base among senior citizens to help them to make informed decisions regarding accessible housing. Additionally increased collaboration, and dialogue between different actors, as well as the adjustment of current laws to stimulate the construction of accessible and affordable housing are among measures that all may be needed to address the current challenges and improve the housing situation for the population ageing in place in a national as well as an international context.

## Data Availability

The data that support the findings of this study are available from the corresponding author on reasonable request.
